# The Early Clinical Outcomes of a Percutaneous Full-Endoscopic Interlaminar Approach via a Surrounding Nerve Root Discectomy Operative Route for the Treatment of Ventral-Type Lumbar Disc Herniation

**DOI:** 10.1155/2018/9157089

**Published:** 2018-02-12

**Authors:** Chao Shi, Weijun Kong, Wenbo Liao, Yanxiao Lu, Yao Fu, Hongquan Wen, Qian Du, Fujun Wu

**Affiliations:** ^1^Department of Spinal Surgery, The First Affiliated Hospital of Zunyi Medical College, Zunyi 563000, China; ^2^Department of Orthopaedic Surgery II, The First Affiliated Hospital of Henan University, Henan 475000, China

## Abstract

The objective of this study is to introduce a method using a percutaneous full-endoscopic interlaminar approach via a surrounding nerve root discectomy (SNRD) operative route that involves removing the protrusive disc via both the shoulder and the axilla of the corresponding nerve root for the treatment of ventral-type lumbar disc herniation (VLDH) and its early clinical symptoms. Twenty-two patients with VLDH satisfied the inclusion criteria and underwent the full-endoscopic interlaminar approach operation via a SNRD successfully during the period from November 2014 to June 2016. All operations were completed without conversion to other surgical techniques. The average operation time was 78.64 ± 25.97 min (50–145 min). The average removed disc tissue volume was 2.87 ± 0.48 ml (2–3.6 ml). No nerve root injury, infection, or other complications occurred. The postoperative ODI and VAS values of low back and sciatic pain were significantly decreased at each time point compared to preoperative measurements (*P* < 0.05). The MacNab scores at the 12-month follow-up included 15 excellent and 7 good scores. In summary, a percutaneous full-endoscopic interlaminar approach through SNRD is a safe and effective treatment option for patients with VLDH.

## 1. Introduction

Percutaneous endoscopic lumbar discectomy (PELD) has an advantage of providing minimally invasive operative procedures, and this effective treatment has recently made great progress in many areas for the treatment of lumbar disc herniation (LDH) [1, 2]. Two different operative approaches exist: transforaminal and interlaminar. The transforaminal approach has been deemed a standard procedure for the treatment of LDH because of its excellent results in terms of nerve root decompression and low complication rates [3]. However, use of the transforaminal approach at the lower lumbar level can be limited due to anatomical constraints, such as a high iliac crest, narrow neuroforamen, and hypertrophied facet joints [3, 4]. The interlaminar approach (ILA) was proposed by Ruetten et al. to avoid the limits of transforaminal access and to obtain the advantages of PELD [5].

According to the primary position of the protrusive disc with a compressed nerve root on axial MRI, lower LDH can be divided into three types: axilla, shoulder, and ventral. Currently, the ILA mainly involves two different operative routes, via the shoulder or axilla, which are both accessed via a single side of the corresponding nerve root. The axilla approach is often chosen for axilla-type protrusion, while the shoulder approach is frequently selected for shoulder-type and ventral-type protrusions [4, 6]. However, Tonosu observed that excessively compressing the nerve root during the manipulation of the extruded fragments via a single side of the corresponding nerve root may lead to complications such as persistent numbness, transient muscular weakness, and transient bladder and bowel disturbance. He also suggested that a ventral-type operation via the shoulder should be attempted to localize the extruded nucleus in the axilla area [7]. Choi et al. considered incomplete removal of a herniated disc to be one of the most important reasons for unsuccessful PELD [8].

To reduce surgery-related complications, we demonstrated that ventral-type lumbar disc herniation (VLDH) can be treated using an ILA to PELD to remove the protrusive disc via the shoulder and the axilla using operative routes of the corresponding nerve root, which we named surrounding nerve root discectomy (SNRD). Using this approach, we have successfully treated 22 cases of VLDH, all of which obtained good results. The purpose of this study is to introduce this method and report our experience.

## 2. Materials and Methods

### 2.1. Patient Characteristics

**Figure 1:**
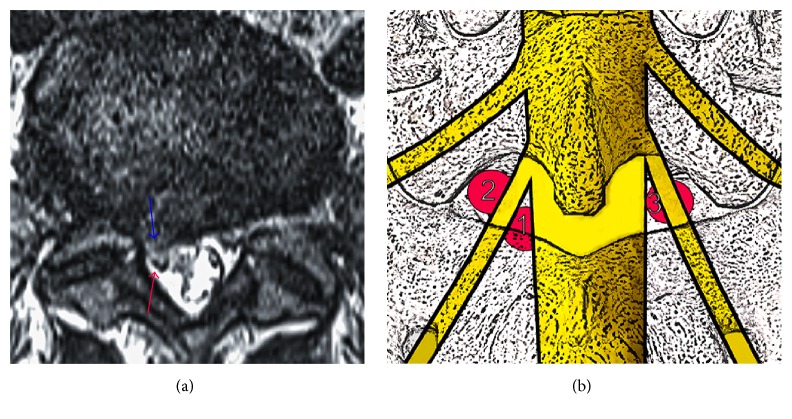
The axial MR image and schematic depiction of ventral-type lumbar disc herniation (VLDH).* Note.* (a) The protrusions are mainly located in the ventral nerve root. The blue arrow indicates the protrusive disc tissues, and the red arrow indicates the nerve root. (b) Lower LDH can be classified into three types: (1) axilla, (2) shoulder, and (3) ventral.

Twenty-two patients (sixteen males, six females) with VLDH (Figure 1) according to the direction of herniation on axial MRI, who were scheduled for a percutaneous full-endoscopic interlaminar approach for SNRD between November 2014 and June 2016, were recruited for this study. Their ages ranged from 18 to 74 years (mean: 40 years), and the duration of symptoms ranged from 1 month to 13 months (mean: 4 months). They received conservative treatments for at least one month and had no obvious symptom alleviation. All patients were subjected to examination of the lumbar disc on lateral X-ray films, CT, and MRI. The protrusive disc was located at L4/5 (8 cases) or L5/S1 (14 cases). The inclusion criteria were as follows: (i) different degrees of lumbago and unilateral obvious sciatica as well as leg pain that was more severe than the waist pain; (ii) the effects of conservative treatment would not last more than 4 weeks; (iii) CT and MRI results showing a single segment of lumbar intervertebral disc herniation with ventral-type protrusion that were consistent with neurological symptoms; (iv) no history of segmental lumbar surgery; and (v) patient informed consent [3, 6]. The exclusion criteria were as follows: (i) an imaging study suggesting lumbar spine stenosis or segmental instability or the diameter of the interlaminar window was less than 8 mm; (ii) central or extreme lateral lumbar disc herniation; (iii) lumbar intervertebral disc with calcification; (iv) a suspected infection or tumour in the lumbar spine; and (v) cauda equina syndrome or another pathological state [3, 6].

### 2.2. Surgical Instruments

A spinal endoscope system (SPINENDOS Co., Germany), including a 4.3 mm working channel, 7 mm outer sheath diameter, and 30-degree angled scope with a continuous water irrigation system and low-temperature radiofrequency ablation system (ArthroCare Co., USA), was used.

### 2.3. Surgical Technique

**Figure 2:**
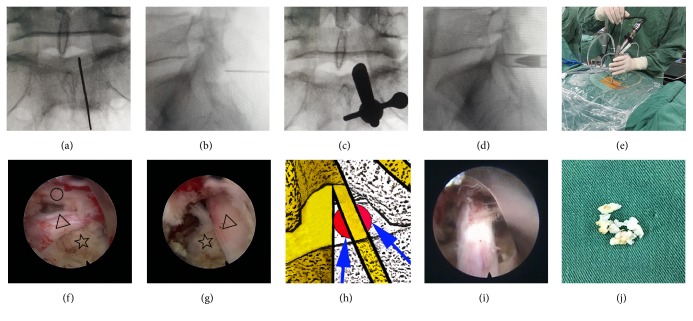
Images of patients with L5/S1 VLDH.* Note.* (a-b) Posterior-anterior and lateral radiography during puncture. (c-d) Posterior-anterior and lateral radiography images showing placement of the tapered, cannulated dilator, and working sheath. (e) Placing the endoscopic system and performing the operation. (f) Insertion of the working sheath into a shoulder region. (g) Insertion of the working sheath into an axillary region with the dural sac gently pushed laterally; the protrusion was in the ventral area of the nerve root. Δ indicates the nerve root, ☆ indicates protrusive disc tissues, and ○ indicates the dural sac. (h) Diagram of the operative route through the shoulder and axillary regions of the nerve root. (i) Removal of the herniated disc material has been completed. (j) The removed disc tissues.

Operations were performed under continuous epidural anaesthesia with patients placed in a prone position on a radiolucent table with their lumbar spine flexed to widen the interlaminar space. This position provided a good anteroposterior radiological view. The line of the vertebral plate gaps was marked by posterior-anterior radiography guidance, whereas the operations were guided by lateral radiography. Once the location of the lumbar segment had been accurately determined, the target position of the puncture was located at the centre of the corresponding interlaminar window. A small skin incision was made, and a tapered cannulated dilator was gently inserted into the target point. Afterwards, the working sheath was inserted via the bevelled opening used for the operation, which was performed under direct visualization, and the site was irrigated continuously with normal saline solution. The ligamentum flavum was incised to allow the endoscope to enter the spinal canal. Radiofrequency (RF) probing was used to manage portions of the adipose tissue and blood vessels. Then, the nerve root, its axilla, and the shoulder area could be exposed. After the insertion of a bevelled opening of working sheath into the axillary region or shoulder region with the nerve gently pushed laterally, a part of the protrusive disc tissues was removed, and the bevelled opening of working sheath was adjusted by placing it on the other region of the nerve root to resect the remaining disc fragment. The nerve root was thus fully decompressed. The nucleus pulposus of the intervertebral disc was subjected to RF ablation. No dural sac damage, significant disc fragmentation, or active bleeding occurred, and good relaxation of the nerve root was noted. Next, all instruments were removed, and the skin incision was closed by a single suture (Figure 2). The operation times, bleeding volumes, removed disc tissue volumes, and intraoperative complications were recorded for each patient.

### 2.4. Follow-Up

Twenty-two patients were examined in follow-up appointments at 1 week and 3, 6, and 12 months after surgery, and each patient received a telephone interview or a questionnaire by mail four working days ahead of their attendance at the outpatient clinic. The follow-up examinations were conducted by two physicians, neither of whom were involved in the operations. In addition to general parameters, other information was obtained using the visual analogue scale (VAS) and the Oswestry low-back pain disability questionnaire (ODI) criteria to evaluate the pre- and postoperative clinical results. Modified Macnab criteria were applied to evaluate the final clinical results.

### 2.5. Statistical Analysis

We performed statistical analyses using the Statistical Package for the Social Sciences (ver. 18.0, SPSS, Chicago, IL, USA). The Tamhane test was applied to compare pre- and postoperative VAS and ODI scores at various times. The results are presented as the mean ± standard deviation. The positive significance level was set at *P* < 0.05.

## 3. Results

**Figure 3:**
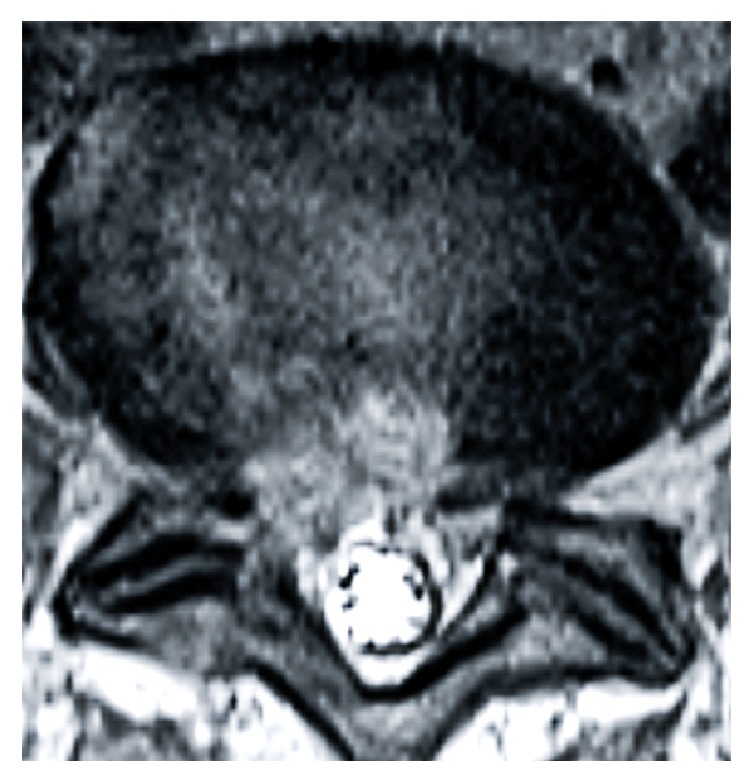
Postoperative MRI results indicating complete removal of the herniated disc material.

All operations were completed successfully; the operation time was 50 to 145 min (78.64 ± 25.97 min); the average removed disc tissue volume was 2.87 ± 0.48 ml (2–3.6 ml), which was measured using a syringe, and no measurable intraoperative blood loss occurred. MRI was conducted to evaluate the completeness of resection of protrusive disc tissue in each patient on day 3 (Figure 3). None of the patients suffered from any postoperative complications, such as blood vessel complications, injury to the nerve or dura, and cerebrospinal fluid leakage or infection. The VAS scores at week 1 (2.27 ± 0.77), as well as at months 3 (1.59 ± 0.59), 6 (0.77 ± 0.53), and 12 (0.59 ± 0.59) after the operation, were significantly decreased compared to the preoperative VAS score (7.32 ± 1.36)  (*P* < 0.05). The ODI scores also showed a significant decrease from 69.53 ± 11.69% preoperatively to 14.79 ± 4.62% at week 1, 11.15 ± 4.98% at month 3, 8.47 ± 3.87% at month 6, and 5.61 ± 2.32% at month 12 after surgery (*P* < 0.05) (Table 1). The curative effect was evaluated with the Modified Macnab criteria after 12 months, and the results indicated excellent outcomes for 15 cases and good outcomes for 7 cases (Table 2).

## 4. Discussion

Due to advances in endoscopic techniques and instrumentation, PELD has become a popular procedure for the treatment of LDH. Many studies have shown good success rates, and some have reported superior outcomes compared to conventional discectomy [9–17]. Two different access points exist for the full-endoscopic technique, including the transforaminal approach and the ILA. PELD was first applied for the treatment of LDH using the YESS system combined with the posterolateral transforaminal approach [18]. However, some factors, such as a high-riding iliac crest (notably at L4/5 and L5/S1) and hyperplastic facet joints, often block the low lumbar segments. The application of the transforaminal approach is limited to removing protrusions of intervertebral discs [3, 4]. Studies of the spinal anatomy have shown that the distance between the edge of the L5 vertebral plate and the L5 vertebral endplate varies from 3.0 mm to 8.5 mm, and the distance between the S1 vertebral plate edge and the S1 vertebral endplate is relatively constant, with an average of approximately 13.9 mm [19], which makes it possible to treat LDH via an ILA. Ruetten et al. proposed this approach to avoid the limits of the transforaminal access while having the advantages of PELD [5]. Afterwards, Ruetten et al. presented good effectiveness of the full-endoscopic technique through an ILA for the treatment of LDH [9, 20, 21].

Nerve roots and the protrusive disc tissues play important roles in achieving successful endoscopic surgery [4]. Based on the location of the protrusive disc with compressed nerve roots on axial MRI, lower LDH can be divided into three types: (i) axilla type, in which protrusions are mainly positioned between the nerve root and dural sac, (ii) shoulder type, in which protrusions are mainly located above the nerve root, and (iii) ventral type, in which protrusions are mainly located in the ventral area of the nerve root. Currently, the ILA mainly includes two different operative routes: (i) via the shoulder with an endoscope and working sheath inserted at the shoulder lateral to the nerve root or (ii) via the axilla, with an endoscope and working sheath placed at the axilla between the nerve root and dural sac. The axilla approach is often chosen for the axilla type, whereas the shoulder approach is frequently selected for the shoulder and ventral type [4, 6]. However, Tonosu observed that excessively compressing the nerve root during manipulation of the extruded fragments via a single side (shoulder approach) of the corresponding nerve root may lead to complications, such as persistent numbness, transient muscular weakness, and transient bladder and bowel disturbance in three cases. He also indicated that the ventral-type operation via the shoulder should sometimes be attempted to localize the extruded nucleus at the axilla area [7]. Choi et al. analysed 10,228 cases to show that the surgical failure rate of PELD was 4.3%. A common cause of failure was incomplete removal of herniated disc material (2.8%) [8]. We also considered that VLDH is unique because the protrusions are neither positioned above the nerve root nor between the nerve root and dural area. The shoulder or a single side of the corresponding nerve root are used to perform discectomy, which may push the nerve root and excessively compress it, leading to incomplete removal of protrusive disc tissues. To reduce such complications, we demonstrate that VLDH can be treated using an ILA for PELD to remove the protrusive disc via both the shoulder and the axilla operative routes of the corresponding nerve root, which we called SNRD. In our study, twenty-two patients underwent this surgery to treat VLDH. All twenty-two operations were successful and proceeded without any complications. The curative effects were thought to be satisfactory, and all patients had significantly reduced postoperative back and leg pain. During the observation period following the operation, no complications related to excessive compression of the nerve root occurred, and postoperative lumbar MRI findings showed no evidence of protrusions compressing the nerves in any of the patients.

We found some factors to be associated with successful surgical outcomes using the ILA for PELD via SNRD, including (i) accurately assessing the anteroposterior and lateral X-ray, CT, and MRI findings before surgery to ensure that the interlaminar window was more than 8 mm to allow direct working channel access to the spinal canal and the pathological segment as well as the protrusive disc tissues that are mainly located in the ventral area of the nerve root and, in some cases like the diameter of the interlaminar window or lateral recess, are relatively narrow; it is to prevent excessively compressing the nerve root and achieve more operating space and have an incomplete removal of an intact disc herniation; we may perform surgery with the descending facet to be drilled; (ii) for VLDH, the operation was performed in the interlaminar centre due to the location of the protrusions and for the convenience of two-side manipulation; (iii) when moving the herniated intervertebral disc from one region to another region by adjusting the working sheath and pushing the nerve root; it is crucial to perform that task slowly, gently, and progressively to prevent excessive compression of the nerve, reduce the risk of dural tears [22], and achieve complete decompression.

In our study, twenty-two patients recovered from the operation satisfactorily and experienced no complications, such as numbness, muscular weakness, and infections. Postoperative sciatica symptoms disappeared, and, most importantly, no obvious recurrence was reported within twelve months of follow-up. We view SNRD as a safe and reasonable supplementation to the ILA for PELD for the treatment of VLDH that offers advantages, such as smaller incisions, reduced tissue damage, adequate nerve root decompression, and reduced complications due to excessive compression of the nerve root during manipulation of the extruded fragments.

### 4.1. Limitations

This retrospective study had some limitations: (i) the sample size was small, and the observation time was short; therefore, a larger sample size and long-term follow-up examinations are needed and (ii) this is not a comparative study but only shows results, and further studies are needed. Despite these limitations, the study results show the usefulness of this technique, and we hope to share it.

## 5. Conclusion

In this study, the results show that a percutaneous full-endoscopic interlaminar approach through SNRD operative route for the treatment of VLDH is a safe and effective surgery option.

## Figures and Tables

**Table 1:** Comparison of functional indicators recorded before performing a percutaneous full-endoscopic interlaminar approach via the surrounding nerve root discectomy operative route and during follow-up (mean ± SD).

Indicators	Pre-op	1 week	3 months	6 months	12 months
VAS	7.32 ± 1.36	2.27 ± 0.77	1.59 ± 0.59	0.77 ± 0.53	0.59 ± 0.59
ODI (%)	69.53 ± 11.69	14.79 ± 4.62	11.15 ± 4.98	8.47 ± 3.87	5.61 ± 2.32

*Note.* VAS and ODI: homogeneity test of variance, *P* < 0.05, single factor analysis of variance using the Tamhane test.

**Table 2:** Grade distribution of the 12-month postoperative effect.

Indicators	Cases	Excellent	Good	Fair	Poor
Modified Macnab criteria	22	15	7	0	0
